# Effects of Body Mass Index, Glycemic Control, and Hypoglycemic Drugs on Serum Uric Acid Levels in Type 2 Diabetic Patients

**DOI:** 10.7759/cureus.3158

**Published:** 2018-08-17

**Authors:** Azhar Hussain, Omar B Latiwesh, Farwa Ali, M.Y. G Younis, Jamal A Alammari

**Affiliations:** 1 Medicine, Xavier University School of Medicine, Oranjestad, ABW; 2 Medical Laboratory, Higher Institute of Medical Professions, Benghazi, LBY; 3 Medicine, American University of Antigua College of Medicine, New York, USA; 4 Assistant Professor and Head of the Department of Biochemistry, University of Benghazi, Faculty of Medicine, Benghazi, LBY; 5 Public Health, Higher Institute of Comprehensive Vocations, Gamins, LBY

**Keywords:** serum uric acid, type 2 diabetes mellitus, cardiovascular diseases, hypoglycemic drugs, insulin

## Abstract

Background

Plasma uric acid has been shown to be associated with an increased risk of hypertension, cardiovascular disease, chronic kidney disease, insulin resistance, and metabolic syndrome. Conflicting data regarding plasma uric acid levels in type 2 diabetes mellitus and their role in the development and progression of diabetic complications have been observed by many studies. The present study aimed to evaluate plasma uric acid levels in type 2 diabetic patients and to determine the effects of hypoglycemic drugs and pharmacologic insulin on plasma uric acid concentration.

Subjects and methods

The study included 162 type 2 diabetic patients divided into three groups (insulin taking group (N=58), glibenclamide taking group (N=40), and metformin taking group (N=64), and 47 normal healthy controls. A questionnaire that included variables such as age, sex, duration of disease, and body mass index (BMI) were answered by all the participants. Blood samples were collected and estimated for serum uric acid (SUA), fasting blood sugar (FBS), and glycated hemoglobin (HbA1c) using standard methods and the data were statistically analyzed.

Results

Diabetic patients showed a significant increase in serum uric acid, fasting blood sugar, glycated hemoglobin, and body mass index when compared to control subjects. The serum uric acid levels of metformin and glibenclamide taking groups were significantly higher than the control group. The difference of serum uric concentration between the insulin taking group and both the control and metformin groups was statistically non-significant. On the other hand, obese diabetics showed a significantly higher serum uric acid than overweight and lean diabetics. Furthermore, serum uric acid had a significant strong positive correlation with body mass index.

Conclusion

Type 2 diabetes* *mellitus (T2DM) is associated with high serum uric acid levels. Hypoglycemic drugs and pharmacologic insulin do not have a large impact on SUA concentration, but obesity seems to be the primary determinant of SUA levels in T2DM patients. The condition of diabetes may have a direct effect on the oxidation of the purine nucleotides resulting in the increased uric acid (UA) levels. In addition, hyperinsulinemia could lead to hyperuricemia by increasing the rate of xanthine oxidase synthesis. There is a strong relationship between T2DM and obesity with high uric acid levels.

## Introduction

Uric acid (UA) is the final oxidation product of purine catabolism [1]. Uric acid can act as a pro-oxidant, particularly at increased concentrations and may thus be a marker of oxidative stress [2]. Thus, it is unclear whether increased concentrations of UA in diseases associated with oxidative stress, such as atherosclerotic coronary heart disease (CHD), stroke, and peripheral arterial occlusive disease, are a protective response or a primary cause [3]. Type 2 diabetes mellitus (T2DM) is a risk factor for nephrolithiasis and has been associated with UA stones [4]. It has been suggested that patients with UA stones, especially if overweight, should be screened for T2DM or metabolic syndrome [5]. The association between high uric acid levels and insulin resistance is not fully understood [3,4]. Hyperuricemia in T2DM is usually the result of underexcretion of urate. Reaven et al. attributed the presence of hyperuricemia in metabolic syndrome to a secondary response to hyperinsulinemia. The association has been attributed to the effects of insulin on proximal tubular urate transport of the kidney [6]. Insulin can also enhance renal tubular sodium reabsorption [7], which in turn can reduce renal excretion of UA. Hyperinsulinemia could lead to hyperuricemia by increasing the rate of xanthine oxidase synthesis, an enzyme involved in UA production [4]. Because endothelial nitric oxide synthase deficiency results in the features of insulin resistance and metabolic syndrome [8], and because UA has been shown to reduce nitric oxide bioavailability [9], consequently, hyperuricemia may have a key role in the pathogenesis of insulin resistance and thus of T2DM. Moreover, many studies have shown the implication of UA and its metabolites in the development and progression of diabetic complications such as peripheral neuropathy [10], retinopathy [11], nephropathy [12], and cardiovascular diseases [13]. T2DM is a major health problem in the Libyan population and accounts for a high mortality rate; hence this study was conducted to evaluate the association of serum UA levels with obesity and glycemic control in T2DM patients and to find out the effects of hypoglycemic drugs and pharmacologic insulin on serum UA levels. This study was presented at the 2nd Libyan Conference on Chemistry and Its Applications, LCCA-2 on September 9, 2017.

## Materials and methods

A total of 162 patients with T2DM were recruited from the Benghazi Center for Diagnosis and Treatment of Diabetes. They are divided into the following three groups according to diabetes treatment: (insulin taking group (N=58), glibenclamide taking group (N=40), and metformin taking group (N=64). Forty-seven apparently healthy age and sex-matched individuals were selected from our family members and relatives to serve as controls. Informed consent was obtained from all the participants before the study, and approval was obtained from the Ethics Review Board of the University of Benghazi.

All patients were diagnosed with T2DM based on the American Diabetes Association criteria 2006, i.e., A1c ≥ 6.5%, or fasting plasma glucose level ≥ 126 mg/dL, or 2-h plasma glucose ≥ 200 mg/dl during an oral glucose tolerance test.

Clinical information and medical history were obtained through a questionnaire that included variables such as age, sex, date of the diagnosis, physical activity, following a diet, and any health problems, or prescribed drugs. The height and weight were measured, and obesity was defined as body mass index (BMI) of ≥ 30 kg/m2, where BMI was calculated by dividing the weight in kilograms over height in meters squared.

All patients presented stable metabolic conditions. Patients presenting any disease that could affect their metabolic status and the parameters studied, such as nephrotic syndrome, acute or chronic renal failure, hepatitis or other liver diseases, cardiovascular diseases, arthritis, acute or chronic inflammatory conditions, gout, and cerebrovascular diseases, were excluded from the study. Patients with a history of smoking or alcohol intake were also excluded. Pregnant and lactating women were excluded. The history of medication was recorded and patients taking any drugs that could affect serum UA levels were also excluded. The control group consisted of healthy subjects who were not suffering from an acute infection or metabolic or psychological disorder. They were non-smokers and non-overweight. They had no history of acquired or inherited hyperuricemia or diabetes mellitus.

Venous blood samples were drawn from all the participants after at least 10 hours of fasting. Blood was collected in ethylenediaminetetraacetic acid (EDTA) and plain tubes, and sera were separated from plain tubes and stored at – 20^o^C until the assays were performed. The whole blood was stored at 4 – 8^o^C and analyzed for HbA1c within a week using a fully automated Cobas Integra 400 plus (Roche, Germany). Sera were analyzed manually for fasting blood sugar, and UA were analyzed by commercial kits supplied by Linear Chemicals SL, Spain, using Photometer 4040v5+ Robert Riele GmbH & Co, Germany.

The data were statistically analyzed using Statistical Package for the Social Sciences (SPSS 17, IBM Corporation). Analysis of variance test (ANOVA) and independent samples T-test were used to determine the variance between different subject groups. Pearson's correlation analysis was done to evaluate the degree of association between different clinical and biochemical parameters. Descriptive characteristics of the study participants were calculated as the mean ± standard deviation (SD), and a P value < 0.05 was considered as statistically significant.

## Results

The mean age and standard deviation of the diabetic group was 51.2 ± 10.9, and the male:female ratio was 4:5. The age range was 18-80 years with the duration of disease ranging from 1-30 years. The mean age and SD of the healthy control subjects was 49.4 ± 12.6, and the male:female ratio was 12:13. The age range was 35-81 years.

As shown in Table 1, body mass index (BMI), fasting blood sugar (FBS), glycosylated hemoglobin (HbA1c), and serum uric acid (SUA) levels were significantly higher in the diabetic group than in the normal control group (p< 0.05). Fasting blood sugar was significantly lower in the control group when compared to each of the diabetic groups (p< 0.05). The difference in FBS was also statistically significant between the metformin taking group and both the insulin and the glibenclamide taking groups (p< 0.05). No significant difference has been found between the insulin group and the glibenclamide group. HbA1c concentration was statistically higher in all diabetic groups when compared to that in the control group (p< 0.05). Moreover, the difference in mean HbA1c between different diabetic groups was statistically significant (p< 0.05). The body mass index was significantly higher in both metformin and glibenclamide groups when compared to either insulin or control groups. On the contrary, significant difference in BMI has not been observed between the metformin group and the glibenclamide group or between the insulin group and the control group. Furthermore, similar to BMI, SUA levels in the metformin and glibenclamide groups were significantly higher than in the insulin and control groups. Similarly, there was a non-significant difference in UA levels between the metformin and the glibenclamide groups, as well as between the insulin and the control groups.

**Table 1:** Mean ± standard deviation (SD) of body mass index (BMI) and biochemical characteristics of type 2 diabetic patients and normal control groups.

Multiple Comparisons (Post Hoc analysis)		Diabetic and control groups					
	Parameters	Metformin (N= 64 )	Glibenclamide (N= 40 )	Insulin (N= 58)	Control (N= 47 )	P value	
	Fasting Blood Sugar (mg/dl)	161.9 ± 58	214.7 ± 91.3	193.6 ± 80	92.7 ± 12	0.00	
	Metformin	-	0.001	0.007	0.00		
	Glibenclamide	0.001	-	0.17	0.00		
	Insulin	0.007	0.17	-	0.00		
	Control	0.00	0.00	0.00	-		
	Glycated Hemoglobin (HbA1c ) (%)	7.7 ± 1.8	9.5 ± 2.4	8.4 ± 2.2	5 ± 0.4	0.00	
	Metformin	-		0.036	0.00		
	Glibenclamide	0.00	-	0.017	0.00		
	Insulin	0.036	0.017	-	0.00		
	Control	0.00	0.00	0.00	-		
	Body Mass Index (kg/ m^2^)	29 ± 6.1	29.4 ± 4.7	26 ± 5.6	24.4 ± 0.5	0.002	
	Metformin	-	0.72	0.004	0.004		
	Glibenclamide	0.72	-	0.012	0.009		
	Insulin	0.004	0.012	-	0.82		
	Control	0.004	0.009	0.82	-		
	Serum Uric Acid (mg/dl)	5.8 ± 2.7	6.4 ± 2	4.9 ± 1.8	4.7 ± 0.9	0.001	
	Metformin	-	0.19	0.02	0.007		
	Glibenclamide	0.19	-	0.003	0.001		
	Insulin	0.02	0.003	-	0.6		
	Control	0.007	0.001	0.6	-		

Dividing diabetic patients according to the BMI cut-off value of 30 kg/m^2^ revealed a significantly higher SUA level in obese diabetics in comparison to lean and overweight diabetics (mean SUA of 6.52 ± 2.6 in obese diabetics versus mean SUA of 5.09 ± 2 in lean and overweight diabetics, p= 0.00).

In addition, by dividing diabetic patients according to the HbA1c cut-off value of 7.5%, we found a significantly higher SUA concentration in patients with HbA1c > 7.5% when compared to those with HbA1c ≤ 7.5% (mean SUA of 6.05 ± 2.5 in diabetics with HbA1c > 7.5% versus mean SUA of 4.9 ± 2.1 in diabetics with HbA1c ≤ 7.5%, p= 0.006).

**Figure 1:**
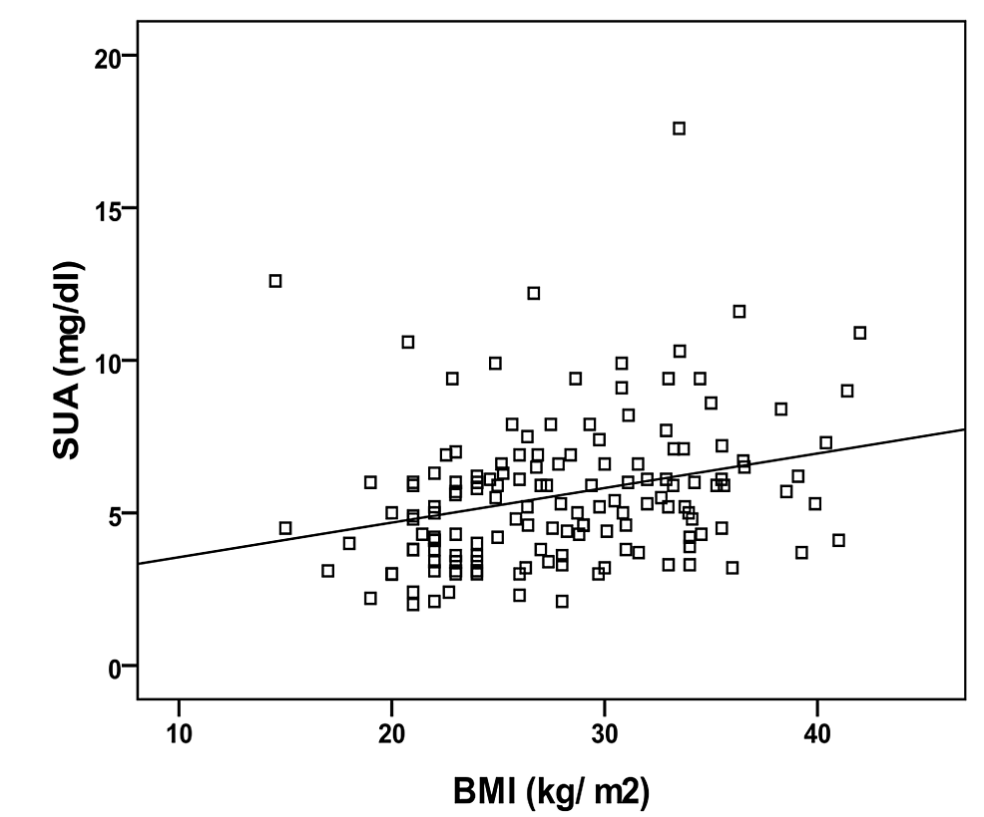
Correlation between serum uric acid (SUA) and body mass index (BMI).

**Figure 2:**
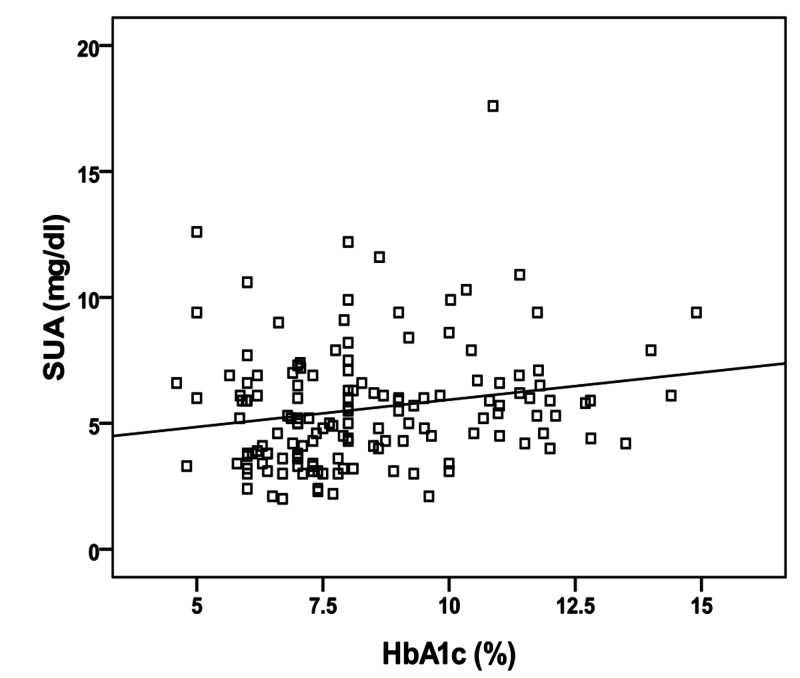
Correlation between serum uric acid (SUA) and glycated hemoglobin (HbA1c).

In the type 2 diabetic group, Pearson’s correlation analysis revealed significantly positive correlations between SUA levels and both body mass index (p= 0.001, r= 0.279) (Figure 1), and HbA1c concentration (p= 0.017, r= 0.197) (Figure 2). On the other hand, no significant correlation has been found between SUA and FBS (p= 0.12, r= 0.126), age (p= 0.5, r= - 0.4), or duration of disease (p= 0.59, r= - 0.05).

## Discussion

In the present case-control study, SUA levels were significantly higher in diabetic patients than normal healthy controls. This finding is in line with data published in previous studies in which high SUA level has been associated with T2DM [14-17].

Persons diagnosed with T2DM have shown very high UA levels in their blood compared to people suffering from diseases such as gout. This indicates that the condition of diabetes may have effects on the oxidation of purine nucleotides. However, the actual relationship between the two is not fully understood due to the complications of metabolic syndrome [17].

Hyperuricemia in T2DM is usually the result of underexcretion of urate as a secondary response to hyperinsulinemia [6,7]. In addition, hyperinsulinemia could lead to hyperuricemia by increasing the rate of xanthine oxidase synthesis, an enzyme involved in UA production [4]. Some studies showed a non-significant difference in UA levels between diabetic patients and controls [18], while other studies showed a significant lower SUA concentration in T2DM patients when compared to normal controls [19].

In the present study, SUA showed significant positive correlation with HbA1c in diabetics. Choi et al. [20] in their study of hemoglobin A1c, fasting glucose, serum C-peptide, and insulin resistance in relation to SUA levels observed that SUA levels and the frequency of hyperuricemia increased with moderately increasing levels of HbA1c and fasting plasma glucose (FPG) and then decreased with further increasing levels of HbA1c (bell-shaped relation). A biological mechanism underlying the bell-shaped relation between blood glucose levels and SUA levels is thought to be due to the uricosuric effect of glycosuria, which occurs when the blood glucose level is greater than 180 mg/dl [21].

We found a significant positive correlation between SUA and BMI in the type 2 diabetic group, and this observation is in agreement with the results of other studies [20-21]. This might be explained with the presence of increased intracellular adenosine (uric acid precursor), a derivative of higher adenosine monophosphate (AMP) concentrations due to increased synthesis of fatty acyl-CoA in peripheral tissues [22], and experiments on mice showed a high xanthine oxidase activity in adipose tissues [23].

In the present study, body mass index was significantly higher in both the metformin and glibenclamide groups when compared to either insulin or control groups. In contrast to our findings, a study by Barskova VG et al., to evaluate results of metformin (MF) therapy during one year of UA metabolism and the clinical course of gout with insulin resistance, revealed the hypouricemic effect of MF and hypothesized that MF reduces the production of UA in patients with gout due to the inhibition of synthesis of free fatty acids [24]. Moreover, metformin use in T2DM improves the sensitivity of peripheral tissues to insulin, which results in a reduction of circulating insulin levels [25], thus decreasing the effect of hyperinsulinemia in reducing the excretion of uric acid. The effect of pharmacologic insulin on SUA has been studied by Lindsey A et al. and Ter Maaten JC et al, and they found a significant increase in serum uric acid levels in diabetic and healthy individuals treated with exogenous insulin, and they referred their finding to insulin’s effects on renal handling of urate [26, 27].

After the treatment with metformin, an increased level of SUA has been observed in type 2 diabetic patients. In comparison to patients treated with rosiglitazone, there is a non-significant difference in SUA levels [28]. A study conducted in Iraq by Ismail NS revealed a non-significant difference in SUA level between both glibenclamide and metformin groups and concluded that glibenclamide and/or metformin had no significant effect on the SUA level in patients with T2DM [29].

In a study by Luque-Ramírez M et al., SUA levels were measured in 40 polycystic ovary syndrome (PCOS) patients and 40 normal healthy women matched for BMI and obesity grade and were followed up for 24 weeks in 34 PCOS patients who were randomized to an oral contraceptive (Diane Diario) or metformin (850 mg twice daily). They found a non-significant difference in SUA between the PCOS group and normal women. When they divided PCOS and normal women as a whole according to BMI, it was revealed that obese women showed higher UA concentrations than lean and overweight women, and that BMI is the main determinant of SUA levels in PCOS patients [30]. This observation supports our finding that metformin and glibenclamide groups both had significantly higher BMI and SUA levels than either insulin group or control group and that SUA level was significantly higher in obese diabetics than lean and overweight diabetics.

## Conclusions

T2DM is associated with high serum uric acid levels. Hypoglycemic drugs and pharmacologic insulin do not have a large impact on SUA concentration, but obesity seems to be the primary determinant of SUA levels in T2DM patients. The condition of diabetes may have a direct effect on the oxidation of the purine nucleotides resulting in increased UA levels. In addition, hyperinsulinemia could lead to hyperuricemia by increasing the rate of xanthine oxidase synthesis. There is a strong relationship between T2DM and obesity with high uric acid levels.
